# Effect of Online Infant Care Education Based on Meleis's Transition Theory on Breastfeeding Success and Discharge Readiness: A Randomized Controlled Trial

**DOI:** 10.1111/ijn.70140

**Published:** 2026-04-12

**Authors:** Fatma Şule Bilgiç, Gülçin Bozkurt

**Affiliations:** ^1^ Faculty of Health Sciences, Department of Midwifery Çanakkale Onsekiz Mart University Çanakkale Turkey; ^2^ Faculty of Health Sciences, Department of Midwifery Istanbul University‐Cerrahpaşa Istanbul Turkey

**Keywords:** breastfeeding, hospital discharge readiness, Meleis, nursing, post‐partum, Transition Theory

## Abstract

**Aim:**

The aim of this study is to examine the effects of online infant care training given to pregnant women, based on Meleis's Transition Theory, on breastfeeding success and readiness for hospital discharge in the postpartum period.

**Background:**

In countries that implement early discharge policies after birth, theory‐based care interventions and training women on infant care during pregnancy are very important.

**Methods:**

This randomized controlled trial was conducted in a public hospital between December and May 2023. The study sample consisted of 102 (intervention group *n* = 52, control group *n* = 50) mothers and infants. Online infant care education based on Meleis's Transition Theory was given to the mothers in the intervention group in the last trimester of pregnancy.

**Results:**

The total breastfeeding success score was 8.88 ± 1.04 in the intervention group and 5.32 ± 1.18 in the control group; the Hospital Discharge Readiness Scale total score was 150.00 ± 19.75 in the intervention group and 133.54 ± 21.28 in the control group. There were significant differences between the intervention and control groups regarding mothers' breastfeeding success scores (*β* = 49.50, *p* < 0.001) and hospital discharge readiness scores (*β* = 756.00, *p* < 0.001).

**Conclusion:**

The findings demonstrate that the intervention significantly enhances breastfeeding success and hospital discharge readiness among mothers. The substantial improvements observed in the intervention group suggest that this approach is an effective and viable strategy for clinical practice. Integrating such structured support programmes into predischarge care is essential for healthcare professionals to optimize maternal and neonatal health outcomes.

## Summary

- Early postpartum discharge is common in many developing countries, including Türkiye, due to limited healthcare resources and workforce constraints.
- Evidence‐based and cost‐effective nursing care models are needed to support maternal and infant health during the early postpartum period.
- An online educational intervention based on Meleis's Transition Theory improves maternal breastfeeding success and readiness for hospital discharge.
- The intervention also contributes to improved postpartum psychological well‐being.
- Theory‐based online education offers a feasible and scalable approach for nurses and midwives to support women during the transition to motherhood.

What is already known about this topic?

- Many developing countries, including Türkiye, adopt early discharge due to lack of capacity and personnel in the postpartum period and economic reasons.
- In these countries, evidence‐based and systematic nursing care models with low personnel and low budgets are needed to protect and improve maternal and child health.
- Meleis's Transition Theory is a well‐established nursing framework used to improve the quality of care during health‐ and life‐related transition periods, including the postpartum period.
- Meleis's Transition Theory is known as a theory that can increase the quality of care for individuals in transition periods of nurses.

What this paper adds?

- Findings demonstrate that an online, theory‐based educational intervention grounded in Meleis's Transition Theory improves maternal breastfeeding success, enhances maternal readiness for hospital discharge and can contribute to postpartum psychological well‐being.
- This paper offers practical, evidence‐based guidance for nurses and midwives to support women during the transition to motherhood.
- This paper highlights the feasibility and scalability of implementing theory‐based online interventions across different healthcare settings.

The implications of this paper?

- This paper suggests that nurses can significantly improve maternal and infant health outcomes by adopting theory‐based care models, providing online education for postpartum mothers and addressing both emotional and practical needs.
- Nurses can play a key role in supporting mothers' transition to motherhood, enhancing breastfeeding success and ensuring discharge readiness, all of which contribute to improved long‐term health for both mothers and their infants.

## 1 Introduction

Motherhood is a learning process that begins with pregnancy and continues after birth, requiring mothers to acquire the knowledge, skills and energy needed to meet the care responsibilities of their newborns (Meighan 2023; Meleis 2010; Öztürk and Erci 2016). Mothers play a crucial role in newborn care, yet they often experience anxiety regarding breastfeeding, infant care and discharge, highlighting the need for information and counselling (Kahyaoğlu Süt et al. 2021). Being prepared for discharge involves more than just leaving the hospital—it includes ensuring that the mother feels confident and empowered, with the opportunity to actively participate in discharge decisions (Erenoğlu and BaŞer 2018).

After discharge, mothers must adjust to the physical and psychological changes of the postpartum period while caring for both themselves and their newborns. First‐time mothers often have concerns about their health and their infant's well‐being during the early weeks (Kahyaoğlu Süt et al. 2021; Karaahmet and Bilgiç 2022). Common challenges during this period include difficulties with breastfeeding and infant care (Karaahmet and Bilgiç 2022).

Breastfeeding, known for its numerous benefits for both mother and infant, is fundamental in infant care (Kılıç 2021). Although breastfeeding has a significant impact on reducing morbidity and mortality rates, global breastfeeding rates remain suboptimal (UNICEF 2019).

Numerous maternal and infant‐related factors influence breastfeeding success (Bilgiç et al. 2023; Yas et al. 2023). A major challenge is the lack of sufficient professional support during breastfeeding, which hinders mothers' ability to overcome difficulties and maintain breastfeeding (Bilgiç et al. 2023; Özkan et al. 2022; Özkan 2021; Durmazoğlu and Okumuş 2019; Yas et al. 2023). Research shows that interventions such as education, counselling, home visits and peer support can significantly improve breastfeeding practices (Yas et al. 2023). Moreover, studies suggest that theory‐based education positively influences breastfeeding attitudes and behaviours (Bilgiç et al. 2023). The relationship between theory and research is reciprocal: research supports theory, and theory, in turn, informs the research process (Ay and Bilgiç 2021). Theoretical frameworks provide a structured approach to research, setting standards for practice and helping to synthesize knowledge, ultimately improving education and care practices (Dündar and Gerçek 2020; Şişman and Arslan 2020; Dağcı 2019).

The inadequacy of prenatal education among women in Turkey is due to the general low level of health education and awareness. Various studies attribute the inadequacy of prenatal education to social, cultural and economic factors. One of the most important factors limiting women's access to prenatal education in Turkey is inequalities in access to health services. Women, especially those living in rural and disadvantaged areas, have limited access to health centres (Özdemir et al. 2020; Burucu and Akın 2017). In addition, gender roles and traditional family structures also affect women's health‐related decisions and may prevent them from receiving prenatal education (Yılmaz and Erdem 2021).

Educational interventions are an effective way to prepare mothers for infant care and parenthood. Online educational programmes, in particular, offer accessibility, cost‐effectiveness and convenience (Ahmed et al. 2016; Sawyer et al. 2017; Zibellini et al. 2021). However, incorrect practices and a lack of knowledge can delay early diagnosis and treatment of infant health issues, adversely affecting their well‐being. Therefore, educating mothers about infant care and health protection starting in the prenatal period is essential. Nonetheless, research on the impact of prenatal education on maternal and neonatal outcomes remains limited (Zibellini et al. 2021).

Meleis (2010) defines nursing as both a science and an art that promotes health and well‐being during transitions. One of its core goals is to ensure healthy transitions, particularly for mothers in the postpartum period. Identifying supportive factors during these transitions allows healthcare professionals to reinforce them and address any obstacles (Şenol et al. 2017; Barimani et al. 2017). In Turkey, early postpartum discharge, insufficient discharge preparation and inadequate support and counselling for mothers are significant challenges. The intervention in this study, based on Meleis's Transition Theory, differs from typical education programmes by focusing not only on the acquisition of practical knowledge but also on the psychological and emotional aspects of the transition to motherhood. Unlike standard infant care education, which primarily addresses caregiving skills, this intervention emphasizes the personal and identity changes mothers experience during this critical period. According to Meleis (2010), successful transitions involve more than the acquisition of new skills; they also require navigating emotional and social changes associated with new roles. This holistic approach aims to strengthen mothers' self‐efficacy and confidence, which are key factors in successful breastfeeding and postpartum adaptation (Meleis 2010; Schumacher and Meleis 1994).

Meleis's Transition Theory is widely used in nursing and healthcare to understand the process of change individuals undergo during significant life transitions. It highlights that those transitions, such as motherhood, are complex processes that involve changes in roles, relationships and self‐identity. The theory emphasizes the importance of support systems, health education and personal empowerment in managing these transitions.

In this study, Meleis's Transition Theory was applied to guide the online infant care education programme, helping mothers navigate the transition to motherhood. The theory provided a structured framework that addressed not only the acquisition of breastfeeding knowledge and infant care skills but also the psychological, physical and social changes mothers experience during the discharge preparation process. This theory‐based intervention aimed to empower mothers by enhancing their knowledge, confidence and self‐efficacy in breastfeeding, ultimately contributing to better breastfeeding outcomes and a smoother transition into motherhood.

## 2 Method

### 2.1 Aim

The aim of this study was to examine the effects of online infant care training given to pregnant women, based on Meleis's Transition Theory, on breastfeeding success and readiness for hospital discharge in the postpartum period.

### 2.2 Research Hypotheses

Hypothesis 1 *There is a statistically significant difference in breastfeeding success between mothers who received infant care education based on Meleis*'*s Transition Theory during pregnancy and those in the control group*.

Hypothesis 2 *There is a statistically significant difference in readiness for hospital discharge between mothers who received infant care education based on Meleis*'*s Transition Theory during pregnancy and those in the control group*.

### 2.3 Design

The study was conducted as a randomized controlled trial at Haseki Training and Research Hospital in Istanbul between December and May 2023. This study was conducted and reported in accordance with the Consolidated Standards of Reporting Trials (CONSORT) guidelines to ensure transparency and methodological rigour (Grant et al. 2018). It was registered on the International Clinical Trials Registry Platform (Registration Number: NCT05812833). The hospital offers free healthcare services, including prenatal preparation classes, and provides care regardless of ethnicity. Although approximately 90% of the population in Istanbul is Turkish, the hospital also serves individuals from various ethnic backgrounds, including Syrians, Turkmens, Iranians and Uzbeks. Syrians, who form a significant portion of the immigrant population in the region, can receive free healthcare at this hospital and other Ministry of Health‐affiliated hospitals. The socio‐economic level in the region where the hospital is located is lower compared with other parts of the province.

### 2.4 Sample

A sample size calculation was performed to ensure that the study had sufficient statistical power to detect a meaningful effect of the breastfeeding education intervention on breastfeeding success. The calculation was based on a study by Aktürk and Kolcu (2023), which reported a large effect size (Cohen's *d* = 0.54) for the impact of breastfeeding education on breastfeeding success, as measured by the LATCH score.

The LATCH score is a widely used tool to assess breastfeeding effectiveness, and in the Aktürk and Kolcu (2023) study, the intervention group showed an average LATCH score of 8.31 (±1.31), while the control group had an average of 7.60 (±1.28). This difference between the groups was statistically significant, indicating that the intervention had a notable effect on breastfeeding success.

To calculate the sample size for this study, we used the G*Power 3.1.9.2 software, a widely used tool for power analysis. The following parameters were used in the calculation:

- Effect size: Based on the results from Aktürk and Kolcu (2023), we adopted a medium‐to‐large effect size of 0.54.
- Alpha error margin: A standard alpha level of 0.05 was chosen, which corresponds to a 5% probability of committing a Type I error (rejecting the null hypothesis when it is actually true).
- Power: A power of 0.80 (80%) was selected, which means that there is an 80% chance of detecting an effect, if one truly exists.

Allocation ratio: Because we plan to have two equal groups (intervention and control), the allocation ratio is 1:1.

With these inputs, the G*Power software calculated the minimum required sample size to be 108 mothers in total, with at least 54 mothers in each group. This ensures that the study would be adequately powered to detect differences in LATCH scores between the intervention and control groups.

By conducting this power analysis, we aimed to minimize the risk of Type II error (failing to detect a real effect) and ensure that our study had sufficient statistical power to support robust conclusions.

**FIGURE 1:**
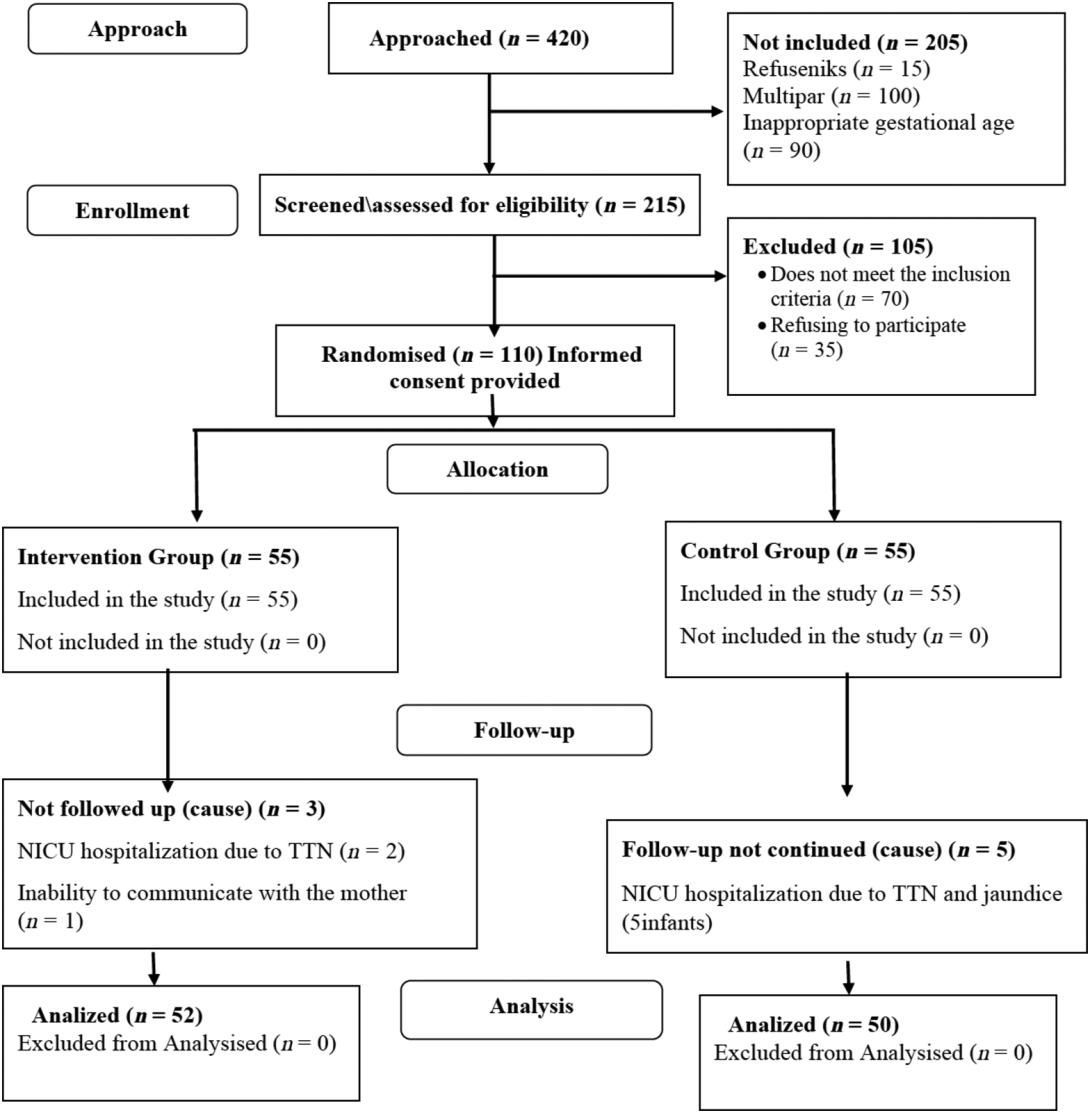
CONSORT flowchart.

To account for potential losses, 110 expectant mothers (55 in each group) were initially recruited. However, three infants in the intervention group and five infants in the control group were excluded due to being transferred to intensive care. The final sample consisted of 102 mothers, with 52 in the intervention group and 50 in the control group (Figure 1).

**Inclusion criteria: women who:**

- Were primiparous (multiparous women were not included in the study because mothers with previous experience could affect the homogeneous structure of the groups and the results)
- Were 32–38 weeks pregnant when first assigned to the training and control groups.
- Were carrying a single pregnancy.
- Had access to the internet and digital devices (smartphone or computer)
- Had not attended prenatal education classes

**Exclusion criteria: women who had:**

- Communication problems
- Chronic maternal health conditions.
- Breastfeeding difficulties (either maternal or neonatal)
- Newborns with Intrauterine Growth Restriction or genetic diseases.
- Not participated in training sessions.

During the follow‐up phase of the study, mothers of infants who were not placed with their mothers after birth for various reasons and who were not born at term (37–42 weeks of pregnancy) were excluded from the study.

### 2.5 Randomization and Blinding

Randomization was performed using the simple randomization method via the ‘https://www.random.org/’ website. Expectant mothers were assigned to either the intervention or control group, with the group assignments being coded as A and B. Lot drawing determined which group would receive the intervention. The randomization process was conducted by an impartial researcher, and the distribution was sealed in opaque envelopes to prevent bias.

Due to the nature of the educational intervention, participants and the researcher delivering the intervention were not blinded. However, the outcome assessor who evaluated breastfeeding success using the LATCH scale and the statistician performing the data analysis were blinded to group allocation. Therefore, the study employed an outcome assessor–blinded design.

### 2.6 Measures

#### 2.6.1 Personal Data Form

This form, developed by the researchers, gathered socio‐demographic, obstetric and birth‐related data for both mothers and infants. The form consisted of two parts. The first part covered the period before the intervention during pregnancy, and the second part, covering the birth and the baby's characteristics, was applied after birth.

#### 2.6.2 LATCH Breastfeeding Charting System

The LATCH scale, validated in Turkish by Yenal and Okumuş (2003), was used to assess breastfeeding success. The scale has five criteria (LATCH) with scores ranging from 0 to 2 for each item. The total score ranges from 0 to 10, with higher scores indicating better breastfeeding success. The Cronbach's alpha value of the scale was found to be 0.95 in the Turkish adaptation study. In this study, the Cronbach's alpha for the LATCH scale was 0.73.

#### 2.6.3 Hospital Discharge Readiness Scale (HDRS)

This study used the Readiness for Hospital Discharge Scale–New Mother Form (RHD‐NMF), originally developed by Weiss et al. (2006) to assess postpartum discharge readiness. The Turkish version of the scale was adapted and its validity and reliability evaluated by Akın and Şahingeri (2010). It includes four subdimensions and a total of 23 items. The first item is binary (yes/no) and not scored, while Items 2–23 are scored on a Likert scale from 0 to 10. A higher score indicates greater readiness for discharge. The subdimensions of the scale are: (1) Personal situation; Items 2–9 (Items 3 and 6 are scored in reverse); (2) Knowledge; Items 10–16; (3) Ability; Items 17–19 and (4) Expected support; Items 20–23. The lowest score that can be obtained from the scale is ‘0’ and the highest score is ‘220’. High scores in scoring indicate that the person's readiness for discharge is sufficient, while low scores indicate that it is not sufficient. In this study, the Cronbach's alpha for HDRS was 0.86.

### 2.7 Procedure

In this study, Meleis's Transition Theory was used as a guiding framework to design and evaluate an online infant care education programme aimed at improving breastfeeding success and hospital discharge readiness in mothers. The theory was particularly relevant because it addresses the transition mothers go through after birth, including adjusting to new roles and responsibilities. The online education programme focused on providing mothers with the knowledge, skills and confidence to successfully breastfeed and care for their newborns, with the goal of facilitating a smoother transition to motherhood. During the education, mothers in the intervention group were divided into groups of 10–12. Each group received two education sessions. All education sessions were interactive. Five experts were consulted to assess the appropriateness of the education content for the study group.

Stage 1: Expectant mothers who met the inclusion criteria were randomly assigned to the intervention and control groups.
Stage 2: Data collection forms were administered to both groups.
Stage 3: Intervention group: Two online group training sessions were held during the antenatal period (32–38 weeks of gestation).

#### 2.7.1 First Session

This session aimed to educate mothers on the importance and benefits of breastfeeding, proper breastfeeding techniques, steps for expressing milk, signs indicating sufficient milk supply, common breastfeeding issues and coping strategies, the importance of infant health monitoring, newborn screenings, vaccinations and the importance of communication between mothers and infants. The session also covered topics such as the structure and benefits of breast milk, reasons for expressing milk and how to store expressed milk. PowerPoint presentations, breast and infant models and milk pumps were used during the session, which lasted approximately 40 min. At the end of the session, participants were asked to write a letter to their infants and select a lullaby.

#### 2.7.2 Second Session

The second session focused on providing mothers with the skills needed for infant care. It covered topics such as infant clothing and care products, diaper cleaning and rash care, infant bathing, infant massage, infant safety, the features of an infant room, sleep hygiene and measures to prevent falls and accidents. Additionally, common issues like fever, nasal congestion, vomiting, thrush, gas pain and crying were addressed, along with potential solutions. Various tools, including infant bath materials, infant care items, infant models, cloth and cotton, were used. The content was delivered through a PowerPoint presentation, and each practice was explained step‐by‐step by the researcher. Two instructional videos (18 min total) on infant bathing and massage were also shown to participants.

Throughout the study, the researcher maintained weekly contact with the mothers to address any concerns, and the mothers were encouraged to reach out at any time for support. The counselling sessions primarily focused on infant feeding, breastfeeding, diaper care, nasal congestion and crying (see Supporting Information).

#### 2.7.3 Control Group

Mothers in the control group followed routine clinical care. No additional training on infant care was provided. However, the training materials from the intervention group were shared with the control group via WhatsApp in PDF format shortly after enrolment. Both groups received approximately 20 min of breastfeeding training in the postpartum period according to hospital policy.

Stage 4: Breastfeeding success was assessed using the LATCH scale by an impartial breastfeeding midwife who was unaware of group assignments. Readiness for hospital discharge was evaluated before discharge using the HDRS.

### 2.8 Statistical Analysis

The data obtained from the study were analysed using SPSS (IBM Corp., Armonk, NY, USA). Descriptive statistics are presented as numbers, percentages, mean ± standard deviation (mean ± SD) and minimum and maximum values. The normal distribution of the data was examined using the Kolmogorov–Smirnov test, and nonparametric tests were used for variables that were not normally distributed. The Mann–Whitney *U* test was used to compare continuous variables between two independent groups. This test is suitable for assessing median differences between groups and was chosen for data that did not meet the normal distribution assumption. The effect size (*r*) was calculated using the Mann–Whitney *U* test, and the practical significance of the results was also assessed (*r* = *Z*/√*N*). Correlations between categorical variables were examined using the chi‐square test. Fisher's exact test was used when the expected values in the cells were less than 5. The significance level was accepted as *p* < 0.05. Due to the nature of the study, because mothers received training during pregnancy, pretest and posttest measurements were not available. Therefore, analyses were conducted only on postintervention comparisons of the two groups. Mean LATCH scores and mothers' readiness for discharge were compared between the two groups after the intervention using Mann–Whitney *U* and chi‐square tests. The effect size in LATCH scores was calculated. All these analyses were designed to statistically assess the differences between the groups and increase the reliability of the results.

In this study, an intention‐to‐treat (ITT) approach was applied. All participants were analysed according to the groups to which they were originally assigned, regardless of whether they completed the intervention or had missing outcome data. For participants with incomplete data, appropriate methods for handling missing values (e.g., last observation carried forward) were employed to maintain the integrity of group assignment and ensure robust comparisons between groups.

### 2.9 Ethical Aspects of Research

In order to conduct the study, approval was obtained from the Haliç Non‐Interventional Clinical Research Ethics Committee (Date: 04.27.2022; No:84) and the necessary permissions were obtained from the institution where the data was collected. At the beginning of the study, the mothers were told about the purpose and method of the research, and their written and verbal consent was obtained.

## 3 Results

The average gestational age at birth was 39.03 ± 1.02 weeks in the intervention group and 38.78 ± 1.21 weeks in the control group. In terms of mode of delivery, 63.5% of births in the intervention group and 58.0% in the control group were by caesarean section. Statistical analysis showed no significant differences between the groups regarding age, education level, employment status, income, planned family type or mode of delivery. Additionally, there were no significant differences between the groups in terms of gestational age, birth weight, birth length or infant sex (*p* > 0.05; Table 1).

**TABLE 1:** Distribution and comparison of socio‐demographic and obstetric characteristics of mothers and infants according to groups (*N* = 102).

Variable	Intervention group (*n* = 52)		Control group (*n* = 50)		Test value	*p*
	Mean ± SD^a^	(Min–max)	Mean ± SD^a^	(Min–max)		
Mother age	26.35 **±** 3.71	19–35	26.88 ± 4.14	19.00–35.00	1249.50^b^	0.734
Gestation age (week)	39.03 **±** 1.02	37.00–41.00	38.78 **±** 1.21	37.00–41.00	1126.00^b^	0.225
Birth length (cm)	50.75 ± 2.26	42.00–54.00	50.66 ± 1.92	47.00–57.00	1113.00^b^	0.203
Birth weight (g)	3295.61 ± 357.06	2500.00–4000.00	3306.40 ± 334.22	2500.00–4000.00	11283.00^b^	0.909

	*n*	%	*n*	%		
**Education status**						
Primary school	11	21.2	10	20.00	0.290^c^	0.986
High School	16	30.8	16	32.00		
University and above	25	48.1	24	48.00		
**Working status**						
Working	14	26.9	22	44.00	3.255^c^	0.071
Not working	38	73.1	28	56.00		
**Revenue status**						
Income less than expense	13	25.0	15	30.00	0.437^c^	0.804
Income equals expense	26	50.0	22	44.00		
Income is more than expense	13	25.0	13	26.00		
**Family type**						
Nuclear	46	88.5	40	80.00	1.380^c^	0.240
Wide	6	11.5	10	20.00		
**Planned pregnancy**						
Planning	49	94.2	42	84.00	2.773^c^	0.96
Not Planning	3	5.8	8	16.00		
**Type of birth**						
Caesarean	33	63.5	29	58.00	0.319^c^	0.102
Vaginal birth	19	36.5	21	42.00		
**Sex of the infant**						
Girl	27	51.9	17	34.00	3.338^c^	0.68
Boy	25	48.1	33	66.00		

^a^ Standard deviation.

^b^ Mann–Whitney *U*.

^c^ Ki frame analysis.

A significant difference was observed between the groups in mean LATCH scores. The intervention group had a mean LATCH score of 8.88 ± 1.04 (range: 7.00–10.00), whereas the control group had a mean score of 5.32 ± 1.18 (range: 2.00–8.00). This difference was statistically significant (*U* = 49.5, *p* < 0.001) and demonstrated a large effect size (*r* = 0.75), indicating high clinical and practical significance. These findings show that mother–infant care skills were significantly higher in the intervention group compared with the control group (Table 2) and support Hypothesis 1, which proposed a difference in breastfeeding success between mothers who received infant care education based on Meleis's Transition Theory and those who did not.

**TABLE 2:** Comparison of LATCH breastfeeding diagnostic and evaluation scale scores by group (*N* = 102).

Variable	Intervention group (*n* = 52)		Control group (*n* = 50)		Test value	*p*
	Mean ± SD^a^	(Min–max)	Mean ± SD^a^	(Min–max)		
LATCH average score	8.88 ± 1.04	7.00–10.00	5.32 ± 1.18	2.00–8.00	49.500^b^	*p* < 0.001

^a^ Standard deviation.

^b^ Mann–Whitney *U*.

For hospital discharge readiness, the intervention group had a total HDRS score of 150.00 ± 19.75, while the control group had a score of 133.54 ± 21.28. Breaking it down by subdimensions, the mean score for the ‘Personal Status’ subdimension was 56.01 ± 7.79 in the intervention group and 49.18 ± 9.70 in the control group; for the ‘Information’ subdimension, the scores were 53.50 ± 9.84 and 50.48 ± 8.74, respectively; for the ‘Talent’ subdimension, the scores were 23.73 ± 4.30 and 19.76 ± 5.26; and for the ‘Expected Support’ subdimension, the scores were 31.26 ± 6.18 and 26.20 ± 6.53, respectively. Participants' responses to ‘Feeling ready to go home’ did not differ significantly between the groups (*χ* ^2^ = 0.790, *p* = 0.374). However, significant differences were found between the intervention and control groups in terms of HDRS total and subscale scores. The intervention group had a total HDRS score of 150.00 ± 19.75, while the control group had a score of 133.54 ± 21.28 (*U* = 756.0, *p* < 0.001, *r* = 0.52), indicating a medium‐to‐high effect size. Similarly, the HDRS personal status subscale (*U* = 762.0, *p* < 0.001, *r* = 0.48), knowledge subscale (*U* = 1051.0, *p* < 0.001, *r* = 0.25), ability subscale (*U* = 725.0, *p* < 0.001, *r* = 0.45) and expected support subscale (*U* = 757.5, *p* < 0.001, *r* = 0.50) values also showed significant differences between the groups (Table 3). These findings indicate that readiness for hospital discharge was significantly higher in the intervention group and support Hypothesis 2, which proposed a difference in discharge readiness between mothers who received infant care education based on Meleis's Transition Theory and those in the control group.

**TABLE 3:** Comparison of Hospital Discharge Readiness Scale scores by group (*N* = 102).

Variable	Intervention group (*n* = 52)		Control group (*n* = 50)		Test value	*p*
	*n*	%	*n*	%		
**Feeling ready to go home**						
Yes	45	86.5	46	92	0.790	0.374^d^
No	7	13.5	4	8		

Variable	Mean ± SD^a^	(Min–max)	Mean ± SD^a^	(Min–max)	Test value	*p*
HDRS^c^ total score	150.00 ± 19.75	108.00–107.00	133.54 ± 21.28	98.00–172.00	756.000^b^	*p* < 0.001
HDRS^c^ personal status subdimension	56.01 ± 7.79	39.00–72.00	49.18 ± 9.70	29.00–70.00	762.000^b^	*p* < 0.001
HDRS^c^ information subdimension	53.50 ± 9.84	30.00–70.00	50.48 ± 8.74	34.00–65.00	1051.000^b^	*p* < 0.001
HDRS^c^ talent subdimension	23.73 ± 4.30	13.00–30.00	19.76 ± 5.26	7.00–29.00	725.000^b^	*p* < 0.001
HDRS^c^ expected support	31.26 ± 6.18	16.00–40.00	26.20 ± 6.53	13.00–39.00	757.500^b^	*p* < 0.001

^a^ Standard deviation.

^b^ Mann–Whitney *U*.

^c^ HDRS: Hospital Discharge Readiness Scale.

^d^ Chi square.

## 4 Discussion

The aim of this study is to examine the effects of online infant care training given to pregnant women, based on Meleis's Transition Theory, on breastfeeding success and readiness for hospital discharge in the postpartum period. The findings indicated that this education significantly increased both breastfeeding success and mothers' readiness for discharge. These results suggest that incorporating Meleis's Transition Theory into midwifery and nursing interventions during the postpartum transition period can enhance care quality and improve mothers' preparation for breastfeeding and discharge (Konuk and Serpil 2020; Meleis 2010).

Antenatal education is a crucial component of maternal care globally, as it prepares women for the challenges of childbirth, breastfeeding and infant care. It provides women with the necessary knowledge and skills to successfully navigate the transition to motherhood (Çolak and Öztürk 2022). Despite its global significance, antenatal education is not widely provided to women in Turkey, which stands in stark contrast to practices in many other countries. This lack of antenatal education is influenced by a variety of factors, including social, cultural and systemic barriers. Studies have shown that inadequate prenatal education is related to traditional beliefs and low levels of education among women, particularly in rural areas (Ünal et al. 2022; Aydin and Yılmaz 2021).

Globally, antenatal education is often a standard part of prenatal care (Shafiei et al. 2019). Many countries have well‐established programmes that equip women with the knowledge they need to prepare for birth and postpartum care. In countries such as Australia, Canada and Sweden, where antenatal education is a cornerstone of maternal care, women are typically discharged within 2–3 days after an uncomplicated vaginal birth (Campbell et al. 2016). This relatively short hospital stay makes antenatal education all the more important, as women need to be well prepared to care for their infants and manage breastfeeding as soon as they return home. Early discharge, if not accompanied by adequate education, can lead to difficulties with breastfeeding and infant care, as women may not have had sufficient time to receive critical information and support (Jones et al. 2021; Chipojola et al. 2020).

However, in Turkey, antenatal education is not as widespread, and the lack of structured prenatal care is a significant concern. This issue is compounded by several factors. First, social and cultural attitudes towards pregnancy and childbirth can limit the importance placed on prenatal education. Traditional beliefs and low levels of education often result in women not seeking or receiving the education they need to prepare for childbirth and infant care (Ünal et al. 2022). Second, there is significant inequality in the geographical distribution of healthcare services, particularly in rural areas, where access to formal antenatal education programmes is limited (Aydin and Yılmaz 2021). These factors contribute to a lack of preparation among women, which can negatively impact their breastfeeding success and overall readiness for motherhood (Kılıç and Demirtaş 2023).

The lack of antenatal education in Turkey may also stem from systemic challenges within the healthcare system. Despite advancements in primary care services, such as family medicine, there is still no systematic structure in place for comprehensive pregnancy follow‐up and education. In many cases, pregnancy is viewed as a medical condition rather than a holistic experience that requires preparation and education. This narrow approach limits women's access to critical information and reduces their ability to make informed decisions regarding childbirth and infant care (Erdoğan and Güler 2020). Health professionals' insufficient training and lack of resources in the area of prenatal education exacerbate these challenges (Kılıç and Demirtaş 2023). Importantly, these systemic barriers are commonly encountered in many other developing countries; therefore, such regions may also derive substantial benefit from the implementation of this intervention to bridge the gap in antenatal support.

One of the key findings of this study is the positive impact of Meleis's Transition Theory–based online infant care education on mothers' readiness for discharge. By improving mothers' knowledge and confidence in managing breastfeeding and infant care, such educational interventions can compensate for the lack of traditional antenatal education. In Turkey, where early discharge is common, particularly after caesarean births, Meleis's Transition Theory offers a valuable framework to guide the transition to motherhood. This theory emphasizes the psychological and emotional processes women go through during childbirth and the postpartum period, making it particularly relevant for enhancing care and preparing women for the challenges they face after birth (Meleis 2010; Eyimaya and Tezel 2020). The current study suggests that interventions based on Meleis's Transition Theory can help bridge the gap caused by the lack of antenatal education, improving mothers' preparedness for discharge. However, addressing the systemic issue of inadequate antenatal education should be a primary focus for improving maternal care in Turkey (Erdoğan and Güler 2020; Aydin and Yılmaz 2021).

The absence of sufficient antenatal education in Turkey highlights a critical gap in the healthcare system. If women are discharged early, without the proper knowledge and support, they are left unprepared to manage the immediate postpartum period. This can lead to complications, including difficulties with breastfeeding and newborn care. Therefore, comprehensive antenatal education programmes are essential to ensure that women are well‐prepared for the challenges of motherhood, especially in the context of early discharge policies (Bilgiç et al. 2023; Jones et al. 2021). As shortened postpartum hospital stays are increasingly reported in various healthcare systems, the need for structured antenatal education and discharge preparation is relevant beyond the Turkish context, suggesting broader applicability of this approach.

In conclusion, this study demonstrates that online infant care education based on Meleis's Transition Theory can play a key role in enhancing breastfeeding success and readiness for discharge. However, to address the underlying issue of inadequate antenatal education, systemic changes are needed in Turkey's healthcare infrastructure. Policymakers should prioritize the development of accessible, comprehensive antenatal education programmes to better prepare women for childbirth and the postpartum period. By doing so, they can improve maternal and infant health outcomes and support women in navigating the critical transition to motherhood (Ünal et al. 2022; Kılıç and Demirtaş 2023).

### 4.1 Limitations and Strengths

One limitation of this study is that it was conducted in a single hospital, which may limit the generalizability of the findings. The study setting may have introduced site‐specific practices, staff characteristics and institutional policies that could have influenced the effectiveness of the online infant care education. Conducting the study in only one centre restricts the applicability of the results to other hospitals or healthcare systems.

Another limitation is related to the cultural background of the participants. All mothers were from a specific cultural context, which could have shaped their perceptions, engagement with the intervention and postpartum behaviours. Cultural norms around pregnancy, breastfeeding and maternal roles may have influenced both breastfeeding success and readiness for discharge, and these factors should be carefully considered when interpreting the findings.

The study design itself is also a limitation. Because the interventions were delivered during the last trimester of pregnancy, pretest and posttest comparisons within groups could not be performed, and only intergroup comparisons were possible. Consequently, intra‐group changes and long‐term effects of the intervention were not assessed.

Despite these limitations, the study's strengths include its theoretical foundation based on Meleis's Transition Theory and the early initiation of training during pregnancy, which offers valuable insights into supporting mothers during their transition to parenthood. Future research should involve multiple hospitals and diverse populations to improve generalizability, systematically evaluate the influence of cultural factors, and include long‐term follow‐up to examine the sustainability of intervention effects. Moreover, studies could further explore how different components of Meleis's Transition Theory contribute to postpartum outcomes across varied healthcare contexts.

## 5 Conclusion

This study demonstrates that online infant care education based on Meleis's Transition Theory has a significant positive effect on both breastfeeding success and discharge readiness. The intervention group that received online education during the last trimester of pregnancy showed a significant improvement in breastfeeding success as measured by the LATCH score. The improved breastfeeding outcomes in the intervention group highlight the importance of providing targeted, theory‐based education during pregnancy to increase mothers' breastfeeding confidence and skills.

In terms of discharge readiness, the intervention group also showed significantly higher scores on the HDRS compared with the control group. The intervention group scored higher on all subscales of the HDRS, including ‘Personal Status’, ‘Knowledge’, ‘Ability’ and ‘Expected Support’. These results suggest that online infant care education based on Meleis's Transition Theory effectively improves mothers' readiness for discharge, and that such education plays an important role in ensuring that mothers are prepared to manage the demands of caring for their newborns after leaving the hospital. Overall, the findings of this study suggest that online infant care education based on Meleis's Transition Theory can significantly improve postpartum breastfeeding success and mothers' readiness for discharge. These results provide valuable evidence for the effectiveness of theory‐based educational interventions in improving key maternal and infant health outcomes, particularly in settings where early discharge is common.

In this study, the online infant care training specifically targeted key transition indicators outlined in the model: ‘transition process’, ‘conditions that facilitate transition’ and ‘response patterns’. By improving mothers' knowledge and skills (cognitive indicators), supporting their self‐confidence and self‐efficacy (psychosocial indicators), and addressing role‐focused preparation for newborn care (role mastery), the programme facilitated a smoother transition to motherhood. These theory‐driven components likely contributed to the observed improvements in breastfeeding success (LATCH scores) and discharge readiness (HDRS total and subscale scores), demonstrating that structured, theory‐based training can effectively support mothers during critical postpartum transition periods.

### 5.1 Implications for Nursing Practice

The results of this study demonstrate that online infant care education based on Meleis's Transition Theory improves both breastfeeding success and mothers' readiness for discharge. This study provides guidance for midwives and nurses to develop more effective educational strategies on infant care and breastfeeding. Additionally, it highlights the importance of supporting mothers not just physically, but also psychologically. Interventions that address emotional challenges such as anxiety, stress and role changes can help alleviate the emotional burden that mothers often face in the postpartum period.

This study enables healthcare professionals to better educate and support mothers and families, contributing to mothers' preparation for parenting and boosting their confidence during this transition. The application of Meleis's Transition Theory in educational and guidance processes can help both mothers and healthcare providers manage this transition more effectively and consciously. Given the hospital population in which this study was conducted, it is believed that these findings, which support a standardized care approach, have the potential to reach a broader population of mothers and infants throughout the country, especially in regions where healthcare personnel shortages exist.

## Author Contributions

**Fatma Şule Bilgiç:** conceptualization, software, validation, formal analysis, investigation, data curation, writing – original draft, writing – review and editing, visualization, supervision, project administration. **Gülçin Bozkurt:** software, validation, formal analysis, investigation, resources, data curation, writing – original draft, writing – review and editing, visualization.

## Funding

The authors have nothing to report.

## Ethics Statement

In order to conduct the study, the Haliç University Non‐Interventional Clinical Research Ethics Committee (Date: 04.27.2022; No:84) and the necessary permissions were obtained from the institution where the data was collected. At the beginning of the study, the mothers were told about the purpose and method of the research, and their written and verbal consent was obtained.

## Conflicts of Interest

The authors declare no conflicts of interest.

## Supporting information




**Data S1:** Supporting Information

## Data Availability

The data supporting the findings of this study are available from the corresponding author upon reasonable request.
