# Supplementary material for: Effect of Online Infant Care Education Based on Meleis's Transition Theory on Breastfeeding Success and Discharge Readiness: A Randomized Controlled Trial
Source: Int J Nurs Pract. 2026 Apr 12;32(2):e70140. doi: 10.1111/ijn.70140 (PMC13071147; doi:10.1111/ijn.70140)
Supplement: Supplementary file 1 — Data S1: Supporting Information [file IJN-32-e70140-s001.docx]

|  | **EDUCATIONAL CONTENT** | | |  |  |
| --- | --- | --- | --- | --- | --- |
| **Aim** | **Content (Topics)** | | | **Teaching Methods and tools** | **Evaluation methods and tools** |
| **FIRST SESSION** | | | | | |
| To explain the importance of breastfeeding and the benefits of breastfeeding,  Explaining breastfeeding technique,  Counting the steps of milking,  Knowing the clues that show that breast milk is sufficient,  Explaining breastfeeding problems and coping methods, | | | Feeding with Breast Milk - Breastfeeding  Structure of breast milk  Benefits of breastfeeding for the baby  Benefits of breastfeeding for the mother  breastfeeding technique  Reasons for expressing breast milk  Steps of expressing breast milk  Storing expressed milk  Tips to show that breast milk is sufficient | Power Point Presentation  breast model,  Baby Model  demonstration | Question and answer (COGNITIVE)  Mother's Self-Confidence Scale (AFFECTIVE),  Readiness Scale for Hygienic Care of the Newborn (SENSORY), LATCH (MOTORIC)  Baby Health Monitoring Form (DEVİNSEL) |
| To explain the importance of baby health monitoring,  Counting newborn screenings and explaining the reasons,  Counting vaccinations and explaining why they are given. | | | Baby Health Monitoring  newborn screenings  vaccinations | Power Point Presentation  Nasal aspirator, degree, clothes, nail clippers, comb, silver cap, silicone nipple, pump |  |
| Explaining the importance of communication between mother and baby | | | Communication between mother and baby | Power Point Presentation |  |
| **SECOND SESSION** | | | | | |
| Knowing the necessary clothing and care products for the baby | | preparation for birth  baby clothes  baby care products | | Power Point Presentation | Question and answer (COGNITIVE)  Mother's Self-Confidence Scale (AFFECTIVE),  Readiness Scale for Hygienic Care of the Newborn (AFFECTIVE), LATCH (MOTORIC)  Baby Health Monitoring Form (DEVİNSEL) |
| Explain how to clean and care for babies' diapers,  He will explain diaper rash care. | | Bottom cleaning and application steps | | Power Point Presentation Baby model, diaper cleaning and care materials, demonstration |  |
| Will explain the steps of belly care application | | Belly care application steps | | Power Point Presentation Baby model, belly care materials, demonstration |  |
| Explaining bathroom safety  Explain the steps of baby bath application,  Baby Massage | | bathroom safety,  Baby bath application steps  Baby massage application steps | | Power Point Presentation Baby model, Baby bathtub and net, baby towel, bath sponge, baby shampoo, soap and diaper rash cream, bath thermometer, baby diaper, Video |  |
| Explaining the importance of baby's safety,  List the features of the baby room,  Explain safe sleeping positions,  Explain protective measures to prevent falls and accidents, | | Baby's Safety  Features of the baby room,  Safe sleeping positions  Preventing falls and accidents | | Power Point Presentation |  |
| He will explain the solutions to common problems (fever, nasal congestion, vomiting, thrush, gas pain, crying, etc.). | | Fever, Nasal congestion, Vomiting, Thrush, Gas pain, Crying | | Power Point Presentation Baby model |  |

| **First session of the training** | | | |
| --- | --- | --- | --- |
| **Goal** | **Content (Topics)** | **Duration** | **Teaching Methods and Tools** |
| 1. To know the necessary clothes and care products for the baby, 2. To explain the importance of breastfeeding and the benefits of breastfeeding, 3. Explain the breastfeeding technique, 4. Counting the steps of milking, 5. To know the clues that indicate that breast milk is sufficient,   Explain the importance of communication between mother and baby   1. Explain the importance of infant health monitoring 2. Counting newborn screenings and explaining the causes, 3. Counting vaccines and explaining why they are given | Preparing for childbirth  Baby clothes  Baby care products  Breastfeeding-Breastfeeding   1. Structure of breast milk 2. The benefits of breastfeeding for the baby 3. Benefits of breastfeeding for the mother 4. Breastfeeding technique 5. Reasons for expressing breast milk 6. Steps for expressing breast milk 7. Storing expressed milk 8. Clues that indicate that breast milk is sufficient   Communication between mother and baby  Infant Health Monitoring   1. Newborn screenings 2. Vaccines | **Total time: 60 min**  Introduction: **7 min**  Preparation for Birth: **5 min**  Breastfeeding Breastfeeding: **20 min**  Mother-Baby Communication: **5 min**  Baby Health Monitoring: **8 min**  Rating: **15 min** | Power Point PresentationIntroduction and use of equipment (Nasal aspirator, degree, clothes, nail clippers, comb, silver cap, silicone nipple, pump)  Breeast Model,  Baby Model |
| **Second session of the training** | | | |
| **Goal** | **Content (Topics)** | **Duration** | **Teaching Methods and Tools** |
| Explain the importance of the baby's safety,  Count the features of the baby's room,  Explain safe sleeping positions,  Explain protective measures to prevent falls and accidents,  Explain how to care for the perineum in babies,   1. Explain the steps of umbilical care application, 2. Explain the safety of the bathroom, 3. Explain the steps of baby bath application, 4. Explain the care of diaper rash.   Explain the solutions to common problems (fever, nasal congestion, vomiting, thrush, gas pains, crying, etc.) | **Baby's Safety**   1. Features of the baby's room, 2. Safe sleeping positions 3. Prevention of falls and accidents   **Baby Care**   1. Steps of applying perineal care 2. Steps of applying belly care 3. Bathroom security, 4. Baby bath application steps 5. Diaper rash care 6. **Common Issues and Recommendations**   Fever, Nasal congestion, Vomiting  Thrush, Gas pain, Cry | **TOTAL Time: 75 min**  Opening**:15 min**  Newborn Safety: **15 min**  Baby Care: **20 min**  Common Issues and Suggestions:**15 min**  Rating: **10 min** | 1. Power Point Presentation Introduction and use of equipment (baby bathtub and net, baby towel, bath sponge, baby shampoo, soap and diaper rash cream, bath thermometer, diaper) 2. Breast Beale,   Baby Model |
